# Colouration mechanism of chrysoprase: insights from colourimetry, spectroscopy and mineralogy

**DOI:** 10.1039/d5ra05339k

**Published:** 2025-09-23

**Authors:** Yuansheng Jiang, Qingfeng Guo, Yu Wang, Vien Cheung, Stephen Westland, Jiayang Han, Xiang Zong, Ying Guo, Dan Wang

**Affiliations:** a School of Gemmology, China University of Geosciences Beijing 100083 China qfguo@cugb.edu.cn; b School of Design, University of Leeds Leeds LS2 9JT UK; c Department of Information and Computing Sciences, Utrecht University Utrecht 3584 CC Netherlands y.wang6@uu.nl; d The Institute of Geology, Chinese Academy of Geological Sciences Beijing 100037 China

## Abstract

Chrysoprase, prized for its attractive apple-green colour, has long intrigued gemologists and mineralogists. Although divalent nickel (Ni^2+^) is clearly established as the chromophore, the specific form and structural state of the Ni-bearing phase remain unresolved. This study investigates the colouration mechanism of chrysoprase by assessing the coupled roles of Ni content and crystallinity and identifying the nature of the Ni host phase. An integrated analytical approach was applied, combining instrumental colourimetry, X-ray diffraction (XRD), X-ray fluorescence (XRF), and near-infrared (NIR) spectroscopy study on ten natural chrysoprase samples spanning pale to vivid green colour, with standardised sample preparation (1 mm double-sided polished slices for colour/NIR, powders for XRD), calibrated instruments, and defined measurement parameters (*e.g.*, 4 cm^−1^ NIR resolution, 0.02° 2*θ* XRD step size). Colourimetric analysis revealed that chroma correlates positively with Ni content and negatively with crystallinity, with Ni itself inversely correlated with crystallinity. As Ni is predominantly hosted in Ni-bearing phyllosilicates, higher Ni contents reflect greater abundances of these phases, where Ni^2+^ enhances chroma *via* optical absorption, and the presence of these phases lowers crystallinity. XRD patterns show a broad basal reflection near *d* ≈ 10 Å, consistent with disordered Ni-phyllosilicates. Moreover, a prominent NIR absorption near 4330 cm^−1^, attributed to Ni–OH vibrational modes, exhibits partial splitting unique to chrysoprase, reflecting a distorted, less hydrated Ni environment compared with pimelite. Together, these observations demonstrate that the green colour of chrysoprase originates from the poorly crystalline Ni-bearing phyllosilicate intermediate between hydrous, disordered pimelite and well-crystallised, anhydrous willemseite. This work clarifies the mineralogical and spectroscopic basis of chrysoprase's colouration, providing a robust explanation for its distinctive colour.

## Abstract 2

Chrysoprase's green colour originates from Ni^2+^ hosted in a poorly crystalline Ni-bearing phyllosilicate intermediate between hydrous pimelite and anhydrous willemseite. Ni content enhances chroma while the phases reduce crystallinity.

## Graphical abstract

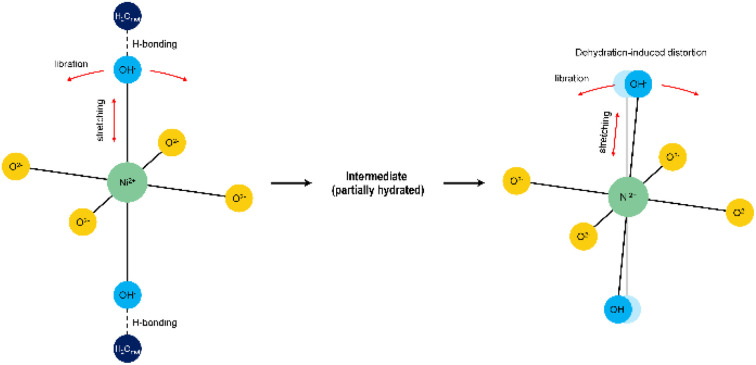

## 1 Introduction

Chrysoprase, known for its distinctive apple-green colour, is a cryptocrystalline quartz variety (chalcedony). Its deposits have been identified in multiple regions worldwide, with Australia being particularly renowned for producing specimens of exceptional quality.^1^ Other notable sources include Brazil, South Africa, Kazakhstan, the United States, Poland,^2^ and Tanzania.^3–5^ The distinctive apple-green colour of chrysoprase sets it apart from other coloured chalcedonies, where Cr^3+^ typically produces intense green (chrome chalcedony)^6^ and Fe^2+^/Fe^3+^ yields red to brown (carnelian).^7^ In contrast, subsequent studies have firmly established that nickel is the key chromophore, with concentrations ranging from trace amounts to several percent by weight.^8^ However, the exact form in which Ni exists within chrysoprase has remained a subject of debate.

Since Ni^2+^ cannot directly substitute for Si^4+^ in the silica lattice, it is widely accepted that the colouration arises from finely dispersed Ni-bearing phases within the silica matrix.^9^ To explain the presence and role of Ni in chrysoprase colouration, two primary models have been proposed. Initially, some researchers attributed the green colour to finely divided bunsenite (NiO),^10,11^ based on evidence such as cubic forms observed in transmission electron microscopy (TEM) micrographs of fractured chrysoprase replicas and a weak reflection at 2.39 Å in the X-ray diffraction (XRD) pattern of chrysoprase from Kazakhstan.^10^ However, this model conflicts with spectroscopic evidence, as the absorption bands in chrysoprase do not correspond to those of NiO.^5,10^ Consequently, attention has shifted toward an alternative model, in which the colouration is linked to finely disseminated Ni-bearing phyllosilicates, including willemseite,^1^ Ni-kerolite,^5,12^ and pimelite,^2,13^ commonly associated with chrysoprase deposits. TEM and XRD observations of layered silicates with interlayer spacings near 10 Å provide strong support for this interpretation.^1,5,12,13^ However, the precise identity of the Ni-bearing phyllosilicates responsible for chrysoprase colouration remains uncertain.

In addition, chrysoprase exhibits marked variation in colour intensity across different samples, suggesting that factors beyond Ni concentration may be involved. As a cryptocrystalline variety of quartz, chrysoprase is likely influenced not only by Ni content but also by structural characteristics such as crystallinity. Previous spectroscopic and microscopic studies have provided valuable insights into possible Ni hosts,^1,5,12–15^ but they have not fully clarified the mineralogical identity of these phases or the mechanistic framework linking chemical composition and structural disorder to colour variation.

Two key knowledge gaps therefore remain: (i) the collective mechanism by which chemical composition (Ni content) and structural disorder (crystallinity) interact to produce the observed colour variation has not been fully resolved, and (ii) the precise mineralogical form and hydration state of the Ni-bearing phases within chrysoprase are still debated. Addressing these questions is essential for establishing a comprehensive model of chrysoprase colouration.

In this study, we combine instrumental colourimetry, XRD, XRF, and NIR spectroscopy to (i) clarify how Ni content and crystallinity jointly govern colour expression, and (ii) determine the mineralogical identity of the Ni-bearing phases in chrysoprase. Although previous studies have established Ni as the chromophore in chrysoprase and suggested that Ni is predominantly hosted within phyllosilicates,^1,5,12–15^ the coupled mechanism linking Ni concentration, structural disorder, and colour enhancement has not been quantitatively resolved. Building on this foundation, we establish a mechanistic framework in which higher Ni contents correspond to greater abundances of Ni-bearing phyllosilicates; the hosted Ni^2+^ ions enhance chroma *via* optical absorption, while the presence of these poorly ordered phases simultaneously reduces crystallinity. Moreover, by integrating NIR and XRD evidence, we identify the colour-causing phase as a poorly crystalline Ni-phyllosilicate intermediate between hydrous pimelite and anhydrous willemseite, thereby resolving a long-standing mineralogical debate. Together, these advances refine the mineralogical and spectroscopic understanding of chrysoprase colouration and fill a critical gap in gemmological research.

## 2 Experimental

### 2.1 Samples

**Fig. 1:**
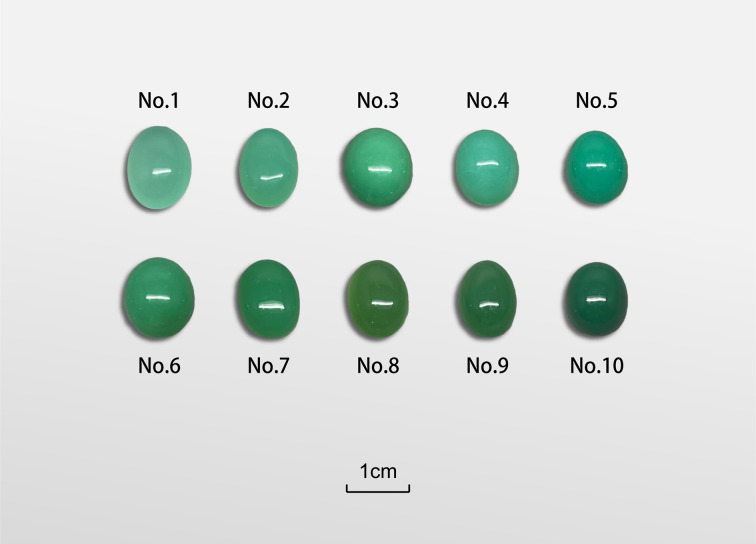
Ten natural chrysoprase samples from Australia showing the natural colour range from light to deep green. Each cabochon is 10 × 15 mm and was later cut into an approximately 1 mm double-sided polished slice for colourimetry, X-ray fluorescence (XRF), and near-infrared (NIR) spectroscopy, with the remaining material ground to 200 mesh for X-ray diffraction (XRD).

A total of ten natural chrysoprase samples from Australia, the most important commercial source of gem-quality chrysoprase worldwide, were selected for this study (Fig. 1). The samples, originally cut as 10 × 15 mm oval cabochons, span a broad colour range from light green to deep green, thereby encompassing the natural variability typically observed in chrysoprase. Each sample was processed into a double-sided polished thin section approximately 1 mm thick, retaining the original oval shape and lateral dimensions, for quantitative colour analysis, X-ray fluorescence (XRF), and near-infrared (NIR) spectroscopy. The remaining material from each sample was ground to 200 mesh for powder X-ray diffraction (XRD) analysis.

### 2.2 CIE (1976) L*a*b*

The CIE (1976) L*a*b* colour space was designed to reflect human visual perception by offering two key advantages: (1) a high degree of perceptual uniformity, meaning that Euclidean distances between colours correspond well to perceived differences, and (2) consistency with the observation that the human eye is less sensitive to differences along the red-green axis than along the yellow-blue axis.^16^ Structurally, CIELAB forms a three-dimensional colour model, where the horizontal plane comprises chromatic coordinates a* and b*, and the vertical axis represents lightness (L*).^17^

Lightness (L*) typically spans from black (0) to white, with higher values indicating a lighter visual appearance.^18^ The a* coordinate reflects the red-green axis, where positive values correspond to red and negative values to green, while the b* coordinate represents the yellow-blue axis, with positive and negative values indicating yellow and blue tones, respectively.^19^ Chroma (C*), describing the intensity of colour, increases from 0 (neutral grey) to higher values representing more vivid colours.^18^ The hue angle (*h*°), expressed from 0° to 360°, identifies the dominant hue along a continuous spectrum of red, orange, yellow, green, cyan, blue, and violet.^19^ All these descriptors are psychophysical quantities and dimensionless. The values of C* and *h*° are derived from the a* and b* values using the following equations:1
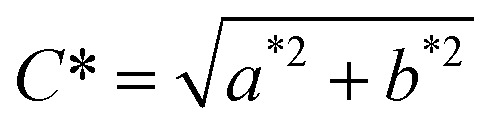
2
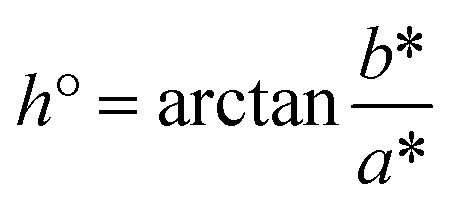


### 2.3 Colour measurement

Colour measurements were performed using an X-Rite i1T Pro spectrophotometer in transmission mode. The instrument was used to record spectral transmittance over the range of 400–700 nm, at 10 nm intervals. The measurements were carried out at a laboratory at No. 18 Longqing Street, Yizhuang Economic and Technological Development Zone, Beijing. The obtained transmittance spectra were used to calculate L*, a*, and b* values under illuminant D65 for the CIE 2° standard observer.

### 2.4 Spearman's rank correlation analysis

To assess the monotonic relationships among several variables, Spearman's rank correlation analysis was performed. This non-parametric method does not require normally distributed data and is particularly suitable for small sample sizes. The analysis was conducted using IBM SPSS Statistics (version 25). For each pair of variables, Spearman's correlation coefficient (*ρ*) was calculated to quantify the strength and direction of the association. The significance of each correlation was tested using a two-tailed hypothesis test. The associated significance (*p*-value) represents the probability of obtaining a correlation as extreme as the observed one under the null hypothesis (*ρ* = 0). Correlations were considered statistically significant when *p* < 0.05. The interpretation of correlation coefficients in terms of strength follows the criteria summarised in Table 1.

**Table 1:** Rule of thumb for interpreting the strength of correlation coefficients^20^

Size of correlation	Interpretation
0.90 to 1.00 (−0.90 to −1.00)	Very high positive (negative) correlation
0.70 to 0.90 (−0.70 to −0.90)	High positive (negative) correlation
0.50 to 0.70 (−0.50 to −0.70)	Moderate positive (negative) correlation
0.30 to 0.50 (−0.30 to −0.50)	Low positive (negative) correlation
0.00 to 0.30 (−0.30 to 0.00)	Negligible correlation

### 2.5 X-ray diffraction (XRD)

X-ray diffraction (XRD) analysis was carried out at Beijing Beida Zhihui Microstructure Analysis and Testing Center Co., Ltd. (Beijing). The measurements were performed using a Bruker D8 ADVANCE diffractometer with a Cu Kα radiation source (*λ* = 1.5406 Å), operated at 40 kV and 40 mA. The scan range was set from 4.0° to 85.0° 2*θ*, with a step size of 0.02° and a counting time of 0.3 seconds per step. A parabolic filter (51-point smoothing) was applied, and the background was subtracted using the 3/1.0 method. The interplanar spacing (*d*) was calculated from the diffraction angle (2*θ*) using Bragg's equation:3
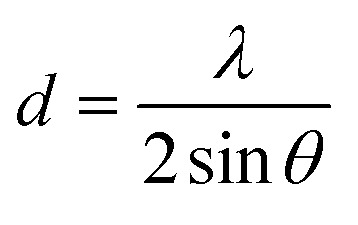
where *d* is the spacing between crystal planes (in Å), *λ* is the X-ray wavelength, and *θ* is the Bragg angle (half of the measured 2*θ* value). In this study, Cu Kα radiation was used with a wavelength of *λ* = 1.5406 Å.

**Fig. 2:**
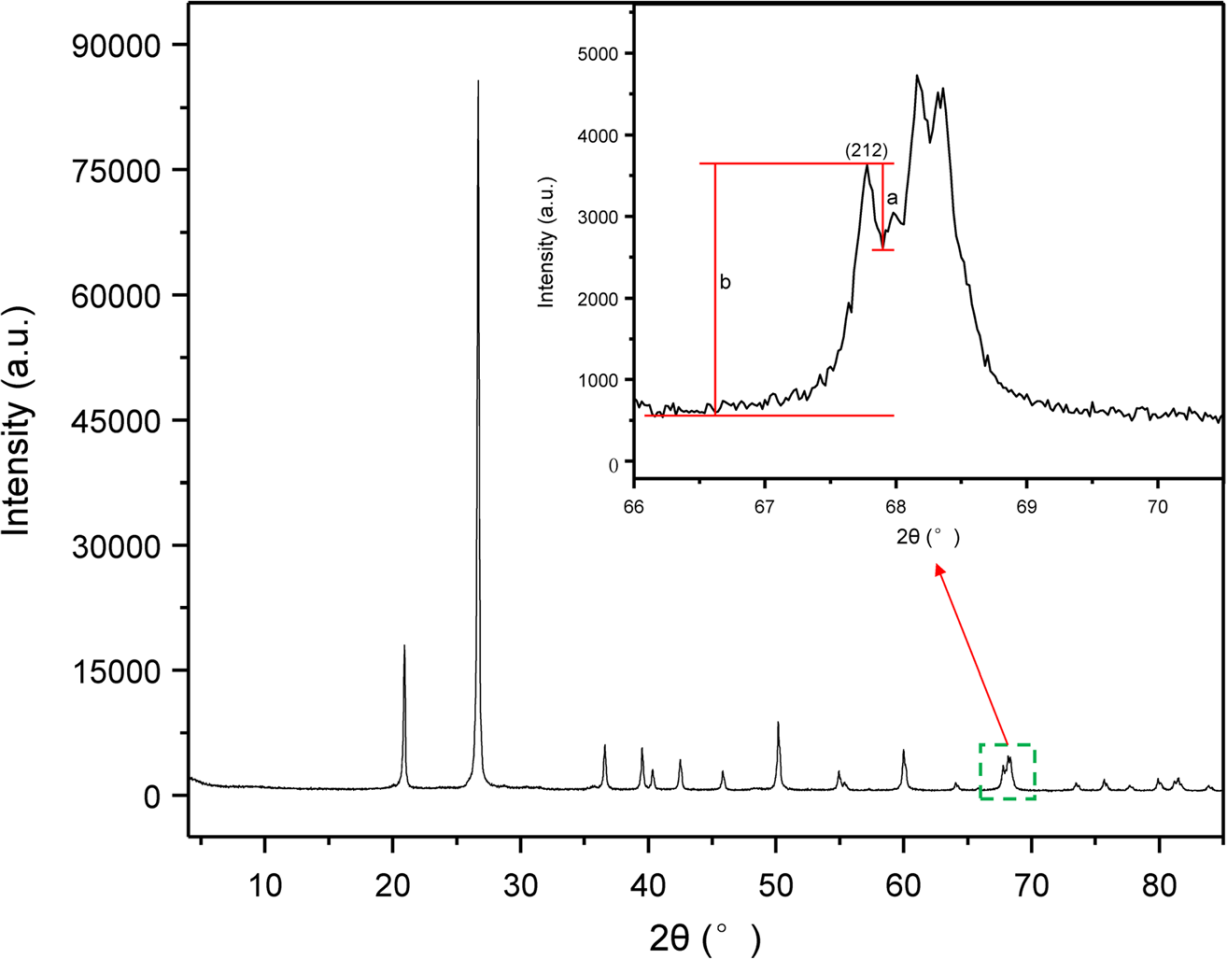
Powder XRD pattern of sample 2 (200-mesh powder). The inset enlarges the (212) reflection at 2*θ* = 67.75°, used to calculate the Crystallinity Index (CI) following Murata and Norman.^21^ Peak height (*a*) and total peak height above background (*b*) are indicated; CI values are normalised using scaling factor *F* = 1.12.

To quantitatively evaluate the crystallinity of the chrysoprase samples, the Crystallinity Index (CI) was calculated based on the method proposed by Murata and Norman,^21^ using the (212) reflection at 2*θ* = 67.75°. The CI provides an arbitrary scale from 1 to 10. It was defined as the ratio of the peak height (*a*) to the total height above the background (*b*) (Fig. 2); as a ratio, the index is dimensionless. The corresponding expression is:4
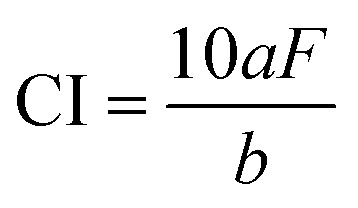
where *F* is a scaling factor used to normalise the CI value to 10 for a synthetic α-quartz standard. In this study, *F* was set to 1.12 based on instrument calibration using this standard material.

### 2.6 Micro X-ray fluorescence (micro-XRF)

The elemental compositions of the chrysoprase samples were analyzed using a Bruker Micro-XRF spectrometer (M4 TORNADO) at the National Infrastructure of Mineral, Rock and Fossil Resources for Science and Technology. The spectrometer is equipped with an X-ray tube (Rh anode) and a polycapillary X-ray optic, which focuses the X-rays onto the sample surface, achieving a spot size of less than 20 μm (Mo Kα). The instrument operated at 50 kV and 600 μA. A vacuum environment was maintained using a vacuum pump, allowing for the detection of light elements down to sodium (Na). For each sample, a surface scan was performed over a selected area to obtain the average elemental composition. X-ray fluorescence signals were collected using a 30 mm^2^ silicon drift detector (SDD), with a count rate exceeding 600 kcps and an acquisition time of 10 ms per pixel. Data processing was carried out using the Mineral Analyzer software provided with the M4 TORNADO system.

### 2.7 Fourier transform near-infrared spectroscopy (FT-NIR)

Near-infrared (NIR) absorption spectroscopy was conducted in the Laboratory of the School of Gemmology, China University of Geosciences (Beijing), using a Bruker Tensor 27 Fourier-transform infrared (FT-IR) spectrometer. Each sample was cut and double-sided polished into slice approximately 1 mm thick for transmission-mode measurements. Spectra were recorded over the range of 8000–4000 cm^−1^ with a resolution of 4 cm^−1^. The scanning speed was set to 10 kHz, and 32 scans were accumulated for each measurement to ensure signal quality and reliability.

To eliminate the influence of sample thickness and ensure comparability across all spectra, absorbance values were converted to absorption coefficients using the following equation derived from the Beer–Lambert law:5
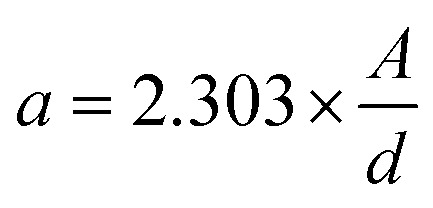
where *a* is the absorption coefficient (cm^−1^), *A* is the absorbance, and *d* is the sample thickness (cm). Baselines for the absorbance bands were determined by drawing straight lines between the two lowest points flanking each band.

## 3 Results

### 3.1 Colour measurement of chrysoprase

The colour data of ten chrysoprase samples are presented in Table 2. The measurements were conducted using a spectrophotometer and expressed in the CIELAB colour space, where L* indicates lightness, a* and b* represent the chromaticity coordinates, C* is the chroma, and *h*° is the hue angle. Simulated colour representations were also generated based on the measured values. It is worth noting that the photographs shown in Fig. 1 represent the samples in their original, unprocessed form, whereas the colour measurements were conducted on 1 mm thick slices. Differences in thickness and shape may cause noticeable discrepancies between the simulated colours and the photographed appearances.

**Table 2:** Measured colour data and crystallinity index of chrysoprase samples

Sample	L*	a*	b*	C*	*h*°	Simulated colour	Crystallinity index
1	91.94	−2.40	2.46	3.44	134.29	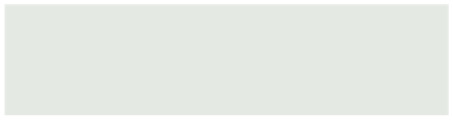	3.01
2	90.45	−3.48	4.02	5.32	130.88	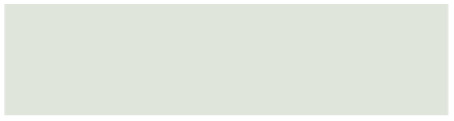	3.90
3	90.26	−6.15	7.15	9.43	130.70	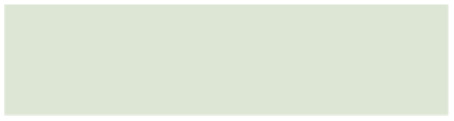	1.69
4	88.31	−4.50	4.31	6.23	136.24	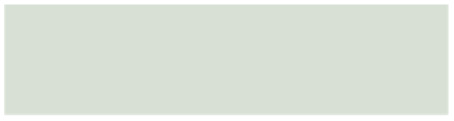	3.67
5	78.43	−10.36	12.03	15.88	130.73	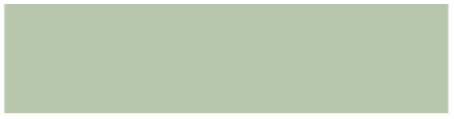	3.36
6	83.57	−8.85	11.65	14.63	127.22	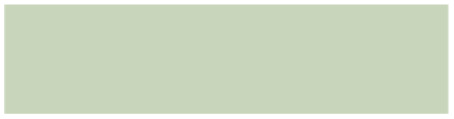	2.45
7	78.64	−11.07	13.95	17.81	128.43	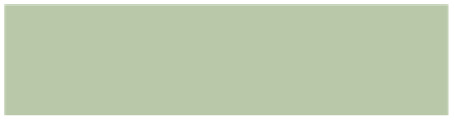	3.15
8	77.12	−18.67	12.72	22.59	145.73	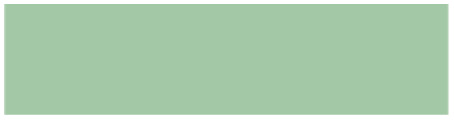	1.57
9	82.13	−11.31	15.41	19.12	126.28	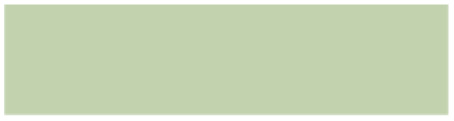	1.94
10	70.91	−22.57	23.13	32.32	134.30	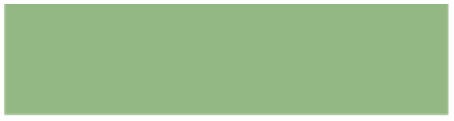	1.22

### 3.2 Elemental concentrations in chrysoprase

As illustrated in Table 3, X-ray fluorescence (XRF) analysis revealed that SiO_2_ is the dominant component in all chrysoprase samples, with concentrations ranging from 93.89 wt% to 99.09 wt%, consistent with the chalcedony nature of the material. The content of NiO, the primary chromophore, varies significantly across the samples, from as low as 0.55 wt% to as high as 5.88 wt%. This wide range indicates notable chemical variability among the samples and suggests a potential influence on colour.

**Table 3:** Element concentrations (wt%) of ten chrysoprase samples determined by XRF

Sample	SiO_2_ (wt%)	NiO (wt%)	MgO (wt%)	CaO (wt%)	Al_2_O_3_ (wt%)	Cr_2_O_3_ (wt%)	Cl^−^ (wt%)
1	99.09	0.55	0	0	0.34	0	0.01
2	98.61	1.02	0	0.01	0.35	0	0.01
3	97.06	1.97	0.60	0	0.33	0	0.04
4	98.18	1.44	0	0.01	0.34	0	0.03
5	96.99	2.60	0	0.01	0.36	0.01	0.03
6	97.45	2.16	0	0	0.34	0.02	0.02
7	97.14	2.44	0	0.01	0.36	0.02	0.03
8	95.61	4.24	0	0	0.11	0	0.03
9	97.47	2.36	0	0.01	0.10	0.02	0.04
10	93.89	5.88	0	0.01	0.10	0.07	0.06

Minor elements such as Al_2_O_3_ and Cr_2_O_3_ are also detected in trace amounts, with Al_2_O_3_ ranging from 0.10 to 0.36 wt% and Cr_2_O_3_ typically below 0.07 wt%. MgO and CaO are nearly absent, appearing only in one sample (sample 3) with MgO at 0.6 wt%, suggesting localized mineral inclusions. Trace chloride (Cl) (≤0.06 wt%) detected in all samples may be associated with minor amounts of hydrous clay minerals, which can incorporate chloride ions through interlayer adsorption or structural substitution.^22^

### 3.3 XRD patterns of chrysoprase

**Fig. 3:**
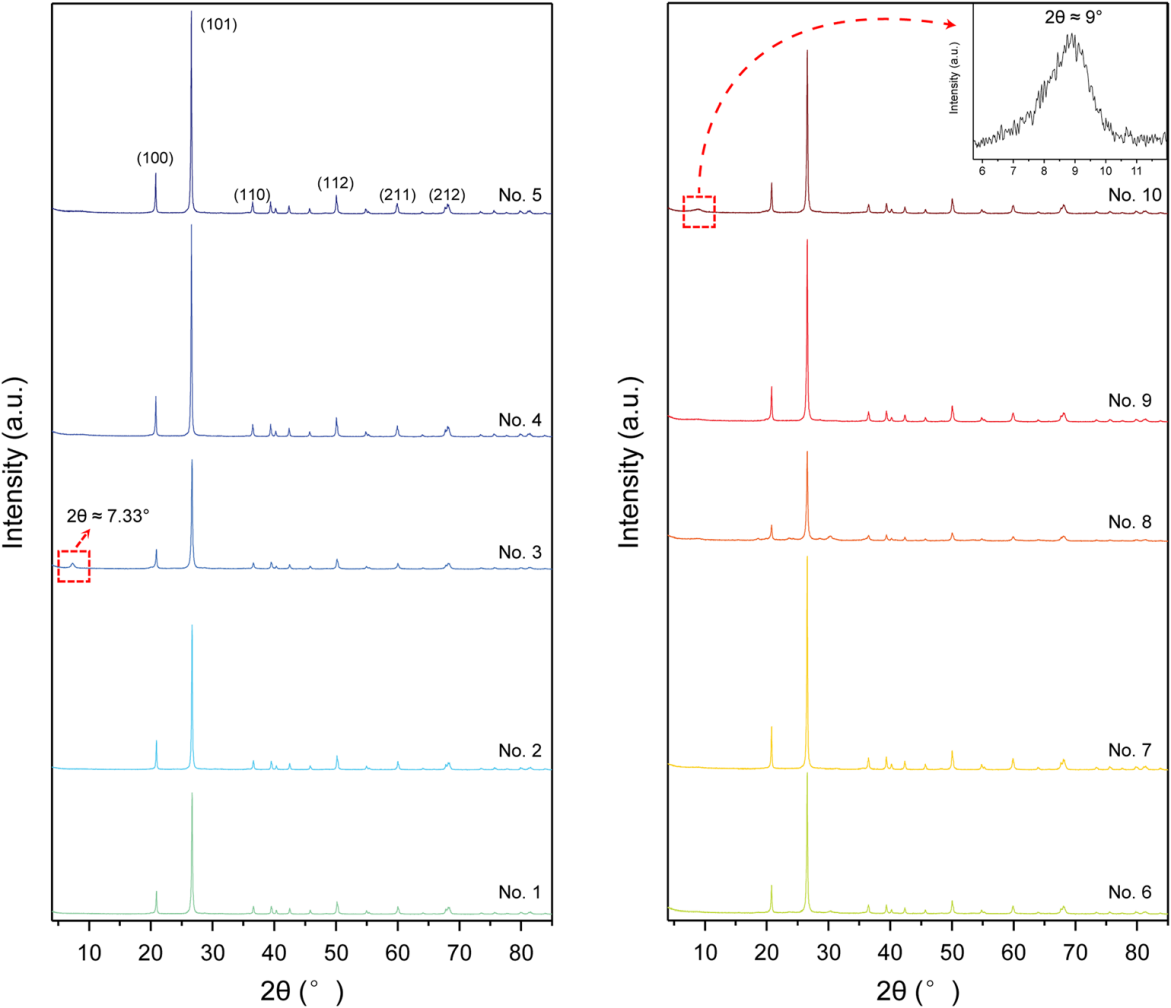
XRD patterns of ten chrysoprase samples, consistent with the α-quartz reference (PDF #86-2237). Minor peaks marked by red boxes indicate possible secondary phases or impurities—for example, the sharp ∼7.33° peak in sample 3 (assigned to sepiolite) and a broad weak reflection near *d* ≈ 10 Å in sample 10, consistent with poorly crystalline Ni-bearing phyllosilicates.

As depicted in Fig. 3, the X-ray diffraction (XRD) patterns of the chrysoprase samples are in good agreement with the standard reference pattern of α-quartz (PDF #86-2237), consistent with the patterns typically observed in other coloured varieties of chalcedony.^23,24^ The most intense reflection appears at 2*θ* = 26.64°, corresponding to the (101) plane, followed by prominent peaks at 2*θ* = 20.86° (100). Additional medium-intensity peaks were observed at 2*θ* = 36.55° (110), 2*θ* = 39.47° (102), 2*θ* = 40.30° (111), 2*θ* = 42.45° (200), 2*θ* = 45.80° (021), 2*θ* = 50.14° (112), 2*θ* = 54.88° (202), 2*θ* = 59.97° (211), 2*θ* = 67.75° (212), and 2*θ* = 68.16° (203). These reflections collectively confirm that α-quartz is the dominant crystalline phase present in all the samples.

However, several minor diffraction peaks not attributable to α-quartz were observed, indicating the presence of secondary phases or impurities. Notably, sample 3 exhibits a sharp peak at 2*θ* ≈ 7.33°, which, combined with its relatively high Mg content, is assigned to sepiolite. A broad weak reflection corresponding to a basal spacing of approximately 10 Å (2*θ* ≈ 9°) was also detected in several samples, most notably in sample 10, suggesting the presence of poorly crystalline, layered Ni-bearing phyllosilicates.^1,2^ Furthermore, small diffraction peaks corresponding to moganite were identified in some samples.

According to eqn (4), the crystallinity index values of chrysoprase samples are presented in Table 2.

### 3.4 NIR absorption spectra of chrysoprase

**Fig. 4:**
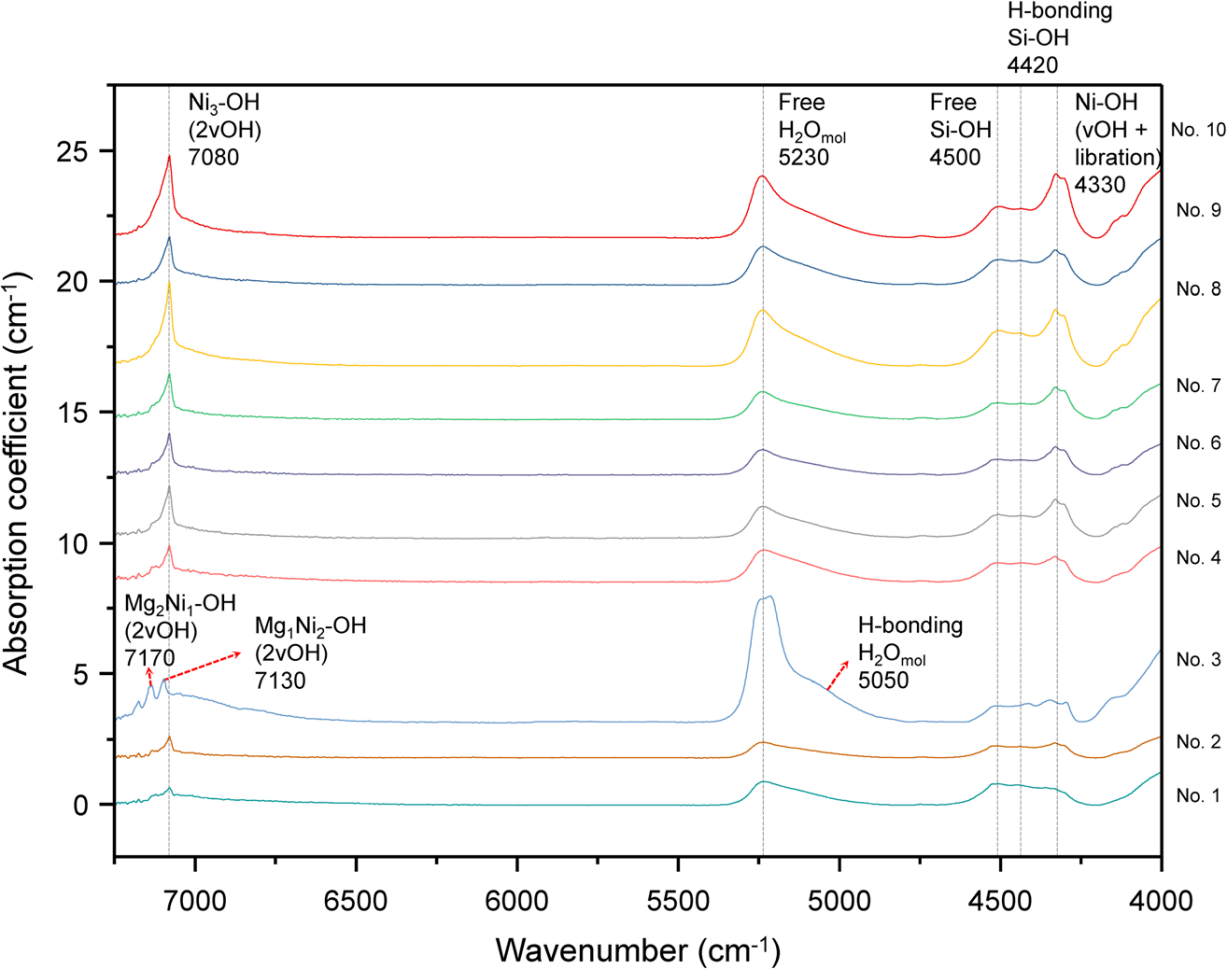
Near-infrared (NIR) absorption spectra (absorption coefficient, cm^−1^) of the ten chrysoprase samples. Key bands are labelled and assigned as follows: ∼7080 cm^−1^ (first overtone of Ni–OH stretching, with minor overlap from Si–OH and H_2_O overtones), ∼5230 cm^−1^ (combination band of non-hydrogen-bonded molecular water), ∼4500 cm^−1^ (isolated Si–OH + siloxane framework vibrations), ∼4420 cm^−1^ (siloxane framework bending + strongly H-bonded germinal silanol), and ∼4330 cm^−1^ (Ni–OH combination band showing partial splitting).

The near-infrared spectra of chrysoprase (Fig. 4) primarily exhibit absorption features associated with Ni–OH groups, silanol (Si–OH) groups, and molecular water. The relative intensities of these bands vary across samples and are summarised in Table 4. Similar Si–OH and H_2_O bands are also observed in other chalcedony varieties, but the distinct Ni–OH absorptions near ∼7080 and ∼4330 cm^−1^ are characteristic of chrysoprase.^25–27^

**Table 4:** Near-infrared absorption coefficients (cm^−1^) of ten chrysoprase samples at characteristic band positions for Ni–OH, Si–OH, and molecular water

Samples	NIR absorption coefficients (cm^−1^, key bands)				
	7080 cm^−1^	5230 cm^−1^	4500 cm^−1^	4420 cm^−1^	4330 cm^−1^
1	0.66	0.91	0.82	0.78	0.62
2	0.80	0.61	0.47	0.44	0.58
3	1.65	4.83	0.63	0.71	0.85
4	1.37	1.23	0.74	0.74	1.00
5	1.98	1.20	0.88	0.84	1.43
6	1.61	0.96	0.59	0.56	1.07
7	1.73	1.03	0.60	0.58	1.20
8	3.20	2.14	1.36	1.26	2.18
9	1.84	1.47	0.98	0.94	1.34
10	3.15	2.37	1.20	1.12	2.45

Water in silica predominantly exists in two forms: free molecular water and silanol groups (Si–OH). Free molecular water may be either non-bonded within structural voids or hydrogen-bonded to silanol groups. Silanol groups, depending on their hydrogen-bonding environment, can occur as free (non-hydrogen-bonded) silanols, water-bonded silanols, germinal silanols (two Si–OH groups sharing the same silicon atom and hydrogen-bonded to each other), or vicinal silanols (hydrogen-bonded to neighbouring silanol groups attached to adjacent silicon atoms). Both the molecular environment and the nature of hydrogen bonding significantly affect the observed infrared absorption features.^26,28–35^

The absorption band centered at approximately 7080 cm^−1^ is primarily attributed to the first overtone (2*ν*_OH_) of the Ni_3_–OH stretching vibration, with minor contributions from the overtone vibrations of silanol groups and molecular water.^36–38^ In addition, the broad band centered at approximately 5230 cm^−1^ is attributed to the combination mode (symmetrical bending plus asymmetric stretching) of molecular water not involved in hydrogen bonding. Furthermore, the absorption band near 4500 cm^−1^ is assigned to the combination vibration of the bending vibration of isolated Si–OH groups and the bending vibration of the siloxane framework (Si–O–Si). The band at 4420 cm^−1^ is attributed to the combination of the bending vibration of the siloxane framework and the stretching vibration of germinal silanol groups with strong hydrogen bonding.^26,34,39,40^ Notably, the sharp absorption feature at approximately 4330 cm^−1^, which appears slightly split, corresponds to a combination vibration involving Ni–OH stretching and librational modes.^41^

According to the XRD and XRF results, sample 3 contains a minor amount of sepiolite (Mg_4_Si_6_O_15_(OH)_2_·6H_2_O). The presence of sepiolite, which contains molecular water, accounts for the enhanced intensity of the 5230 cm^−1^ band in this sample compared to others. Additionally, a shoulder observed near 5050 cm^−1^ is attributed to a combination of symmetrical bending and asymmetric stretching vibrations of hydrogen-bonded molecular water.^26,34,39,40^ Moreover, weak absorptions at approximately 7170 cm^−1^ and 7130 cm^−1^ are assigned to the first overtones (2*ν*_OH_) of the Mg_2_Ni_1_–OH and Mg_1_Ni_2_–OH stretching vibrations, respectively, indicating the presence of mixed Mg–Ni environments around hydroxyl groups.^37,38^

## 4 Discussion

### 4.1 Correlations among colour parameters in chrysoprase

**Fig. 5:**
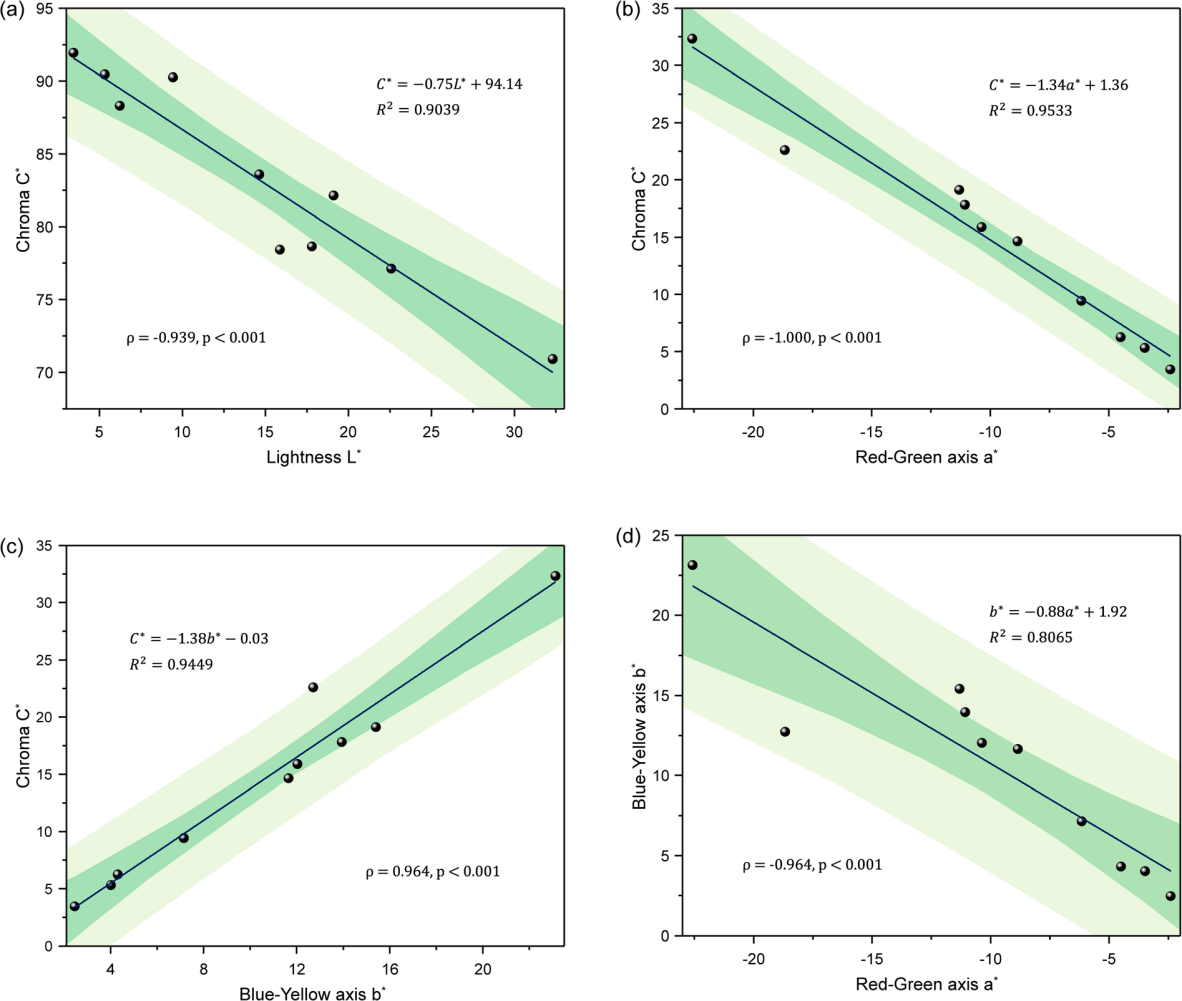
Correlation plots among CIELAB colour parameters of chrysoprase samples. (a) C* vs. L*; (b) C* vs. a*; (c) C* vs. b*; (d) b* vs. a*. Regression lines are shown with 95% confidence intervals (dark green) and 95% prediction intervals (light green). Spearman's rank correlation coefficients (*ρ*), *p*-values, and regression equations are displayed in each plot.

Correlation analysis among the L*, a*, b*, C* and *h*° colour parameters revealed several notable relationships (Fig. 5). A very high negative correlation was observed between lightness (L*) and chroma (C*) (*ρ* = −0.939, *p* < 0.001), indicating that samples with higher chroma tend to exhibit lower lightness. This trend can be attributed to the optical properties of the samples: given their thinness and relatively high transparency, most samples show high L* values overall; however, those with more intense green colouration naturally absorb more light, resulting in reduced perceived lightness.

Additionally, both a* and b* exhibited very high and statistically significant correlations with C*, with *ρ* = −1.000 (*p* < 0.001) and *ρ* = 0.964 (*p* < 0.001), respectively. This result is expected, as chroma (C*) is mathematically derived from a* and b* (eqn (1)). These correlations reflect the inherent mathematical structure of the colour space, rather than independent physical associations.

Moreover, a very high negative relationship was observed between a* and b* (*ρ* = −0.964, *p* < 0.001). This can be explained by the limited variation in hue angles across the dataset: nine of the samples, for instance, displayed hue angles within the narrow range of 126° to 136°, indicating a concentration of hues in the yellowish-green region. This clustering in hue angle suggests that most specimens exhibit a consistent hue direction in the yellowish-green region.

This limited hue variation may also be a consequence of the samples' high transparency and low thickness, which result in generally lower chroma values. When chroma is reduced, colours tend to appear more desaturated or greyish, diminishing the perceptual differences in hue. As a result, a* and b* values tend to vary in tandem along a restricted chromatic pathway, reinforcing their mutual correlation.

### 4.2 Structural and compositional effects on chrysoprase colour

**Fig. 6:**
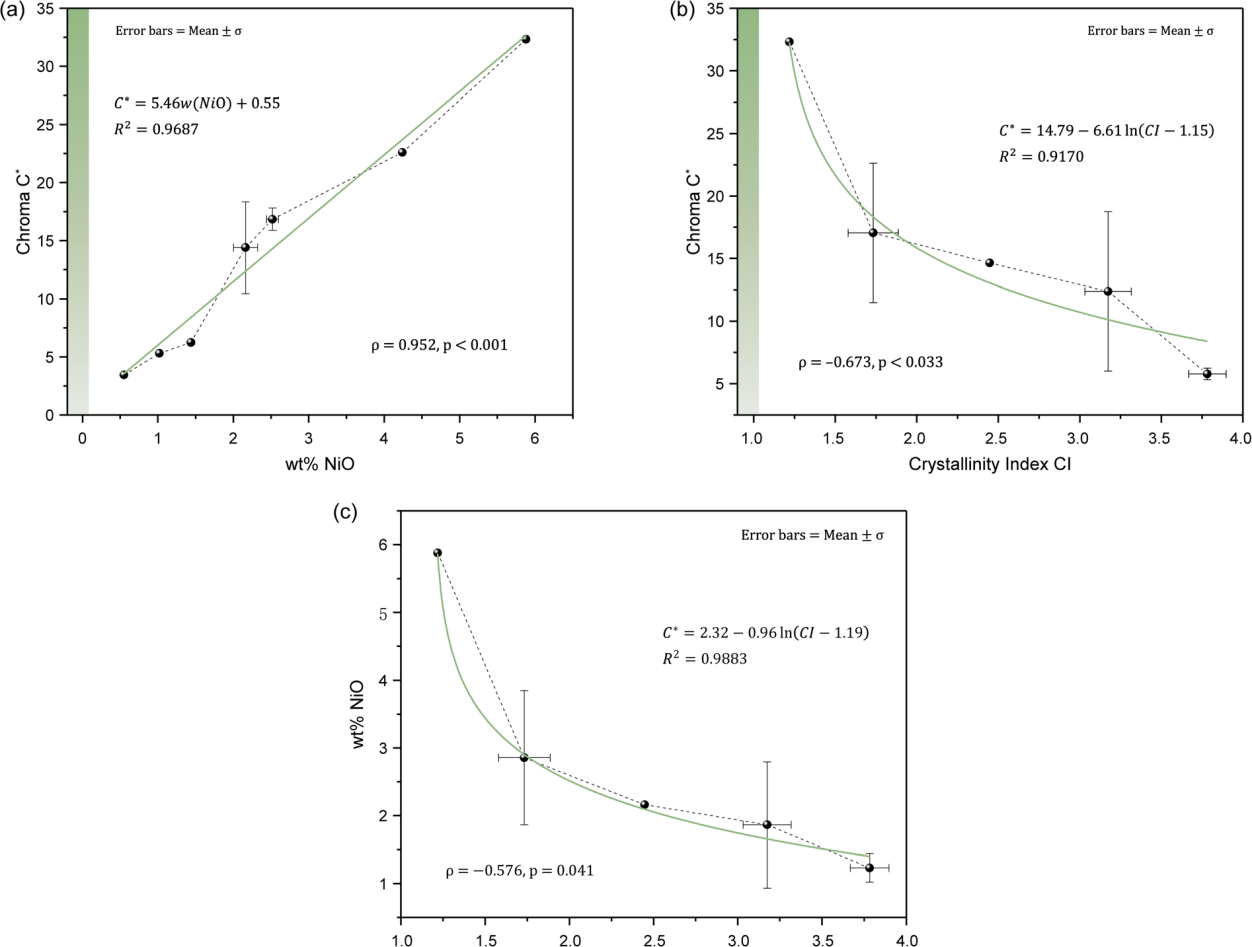
Relationships between chroma (C*) and (a) NiO content, (b) Crystallinity Index (CI), and (c) the relationship between NiO content and CI in chrysoprase samples. Error bars represent mean ± standard deviation. Solid green lines indicate fitted regression models; dotted lines represent overall data trends. A very high positive correlation is observed between NiO and C*, a moderate negative correlation between CI and C*, and a moderate negative monotonic correlation between NiO and CI.

Previous studies have demonstrated that Ni^2+^ is the primary chromophore responsible for the green colouration of chrysoprase.^1,2,4,5,13,42–44^ As shown in Fig. 6a, Ni concentration shows a very strong positive correlation with chroma (C*) (*ρ* = 0.952, *p* < 0.001). Correlation analysis further indicates that Ni concentration is very strongly negatively correlated with L* (*ρ* = −0.988, *p* < 0.001) and a* (*ρ* = −0.952, *p* < 0.001), and strongly positively correlated with b* (*ρ* = 0.891, *p* = 0.001). These findings are consistent with the optical behaviour of Ni^2+^, whose spin-allowed d–d transitions—specifically the ^3^A_2_g(F) → ^3^T_1_g(F) transition—result in enhanced absorption near 660 nm and increased chroma.^13^

Given the established relationships among colourimetric parameters—where chroma is negatively correlated with L* and a* and positively correlated with b*—the increase in Ni content indirectly results in higher b* values and lower L* and a* values.

In addition to chemical composition, the structural order of chrysoprase, as quantified by the crystallinity index, also plays a significant role in colour expression. A moderate negative correlation was found between crystallinity and chroma (C*) (*ρ* = −0.673, *p* = 0.033; Fig. 6b), indicating that lower crystallinity is associated with higher chroma. Since Ni^2+^ cannot directly substitute for Si^4+^ in the silica lattice,^45^ the green colour is interpreted to originate from admixed fine-grained nickel compounds within the silica matrix, rather than from substitutional Ni in the silica structure.^9^ Previous spectroscopic studies, including temperature-programmed reduction (TPR), electron paramagnetic resonance (EPR), and ultraviolet-visible (UV-vis) spectroscopy, have shown that most of the Ni in chrysoprase is hosted in dispersed 2 : 1 phyllosilicates structurally similar to talc.^14,15^ Consistent with this spectroscopic evidence, transmission electron microscopy (TEM) studies have confirmed the presence of these Ni-rich layered silicates, typically tens of nanometers in size, with basal spacings around 10 Å.^1,5,12,13^ Although TEM observations were not available in the present work, which limits our ability to directly confirm phase identity at the nanoscale, the X-ray diffraction (XRD) patterns of several samples show a broad, low-intensity peak near *d* ≈ 10 Å, likewise indicative of the presence of Ni-bearing layered phases. The peak is most prominent in sample 10, which has the highest Ni content. Given that Ni in chrysoprase is predominantly hosted in Ni-bearing layered silicates,^14,15^ higher Ni contents correspond to greater abundances of these phases, leading to a more pronounced ∼10 Å basal reflection. In contrast, samples with lower Ni content contain fewer such silicates, resulting in broader and less distinct XRD peaks.

The presence of finely dispersed Ni-bearing phyllosilicates disrupts the structural order of the silica matrix, thereby reducing crystallinity. A higher bulk Ni content therefore reflects a greater proportion of these poorly ordered phyllosilicates intergrown with chalcedony, which lowers the crystallinity index of the silica phase. Consistent with this mechanism, as shown in Fig. 6c, Ni content and crystallinity index exhibit a moderate negative monotonic correlation (*ρ* = −0.576; one-tailed *p* = 0.041).

These results indicate that the vivid green colouration of chrysoprase is primarily driven by elevated nickel content. Ni^2+^, which is predominantly hosted in Ni-bearing phyllosilicates intergrown with the chalcedony matrix, exhibits optical absorption that directly enhances chroma. The observed negative correlation between Ni content and crystallinity index thus reflects an intrinsic coupling between the abundance of Ni-bearing phases and the degree of structural order, linking compositional and structural factors in controlling chroma expression.

### 4.3 Colour-causing phase inferred from the NIR spectrum

The colour of chrysoprase is attributed to a Ni-bearing layered silicate phase with a basal spacing of approximately 10 Å. This phase may correspond to willemseite (Ni_3_Si_4_O_10_(OH)_2_) or its hydrous analogue, pimelite (Ni_3_Si_4_O_10_(OH)_2_·*n*H_2_O). Willemseite and pimelite are structurally and compositionally related phyllosilicates within the talc group, both adopting a 2 : 1 trioctahedral layer structure in which Ni^2+^ occupies octahedral sites between tetrahedral silicate sheets.^36,46,47^ Willemseite typically exhibits well-ordered, crystalline layers with basal spacings ranging from 9.3 to 9.6 Å, consistent with talc-like structures.^48^ In contrast, pimelite incorporates interlayer water, which disrupts the regular stacking of silicate sheets and results in reduced crystallinity or even amorphous textures.^49,50^ The IMA has proposed that pimelite is essentially a hydrated and disordered form of willemseite,^51^ suggesting a structural continuum within the Ni-talc system, ranging from well-ordered willemseite to poorly crystalline, water-bearing pimelite.

Based on the XRD pattern showing a broad reflection near *d* ≈ 10 Å, the mineral phase in question exhibits poor crystallinity. This suggests that it is unlikely to be well-crystallised willemseite, and is more plausibly a hydrous analogue such as pimelite, which commonly displays low crystallinity due to interlayer water.

While the XRD results suggest the presence of a poorly crystalline Ni-bearing silicate, additional insights can be obtained by analyzing the NIR spectral features. Three water-related absorption bands are observed: a broad band at 5230 cm^−1^ attributed to non-hydrogen-bonded molecular water, a band near 4500 cm^−1^ associated with isolated silanol groups, and a band at 4420 cm^−1^ linked to germinal silanol groups with hydrogen bonding. However, since chrysoprase is primarily composed of cryptocrystalline SiO_2_, which inherently contains both molecular water and silanol groups, the total water-related absorption cannot be used as a reliable criterion to distinguish between willemseite and pimelite. Both phases may contribute overlapping features in the NIR region, making it difficult to definitively attribute these bands to a specific Ni-silicate phase based solely on water content.

**Fig. 7:**
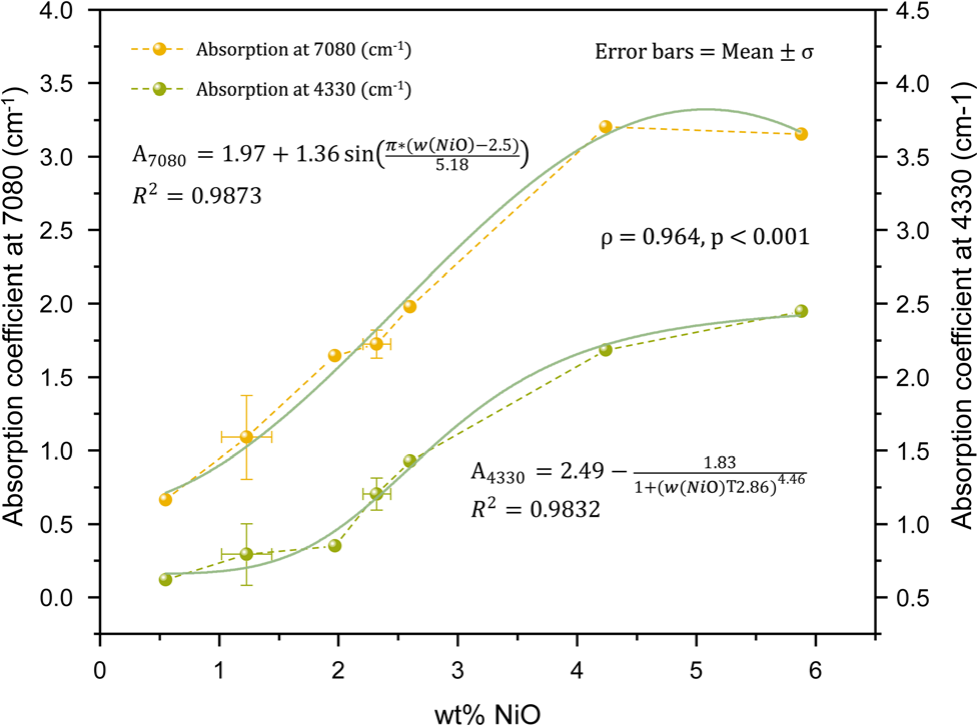
Correlation between NiO content and NIR absorption coefficients at 7080 cm^−1^ and 4330 cm^−1^ in chrysoprase samples. Both bands, attributed to Ni–OH vibrational modes, show very strong positive correlations with Ni content (*ρ* = 0.964, *p* < 0.001). Error bars represent mean ± standard deviation.

**Fig. 8:**
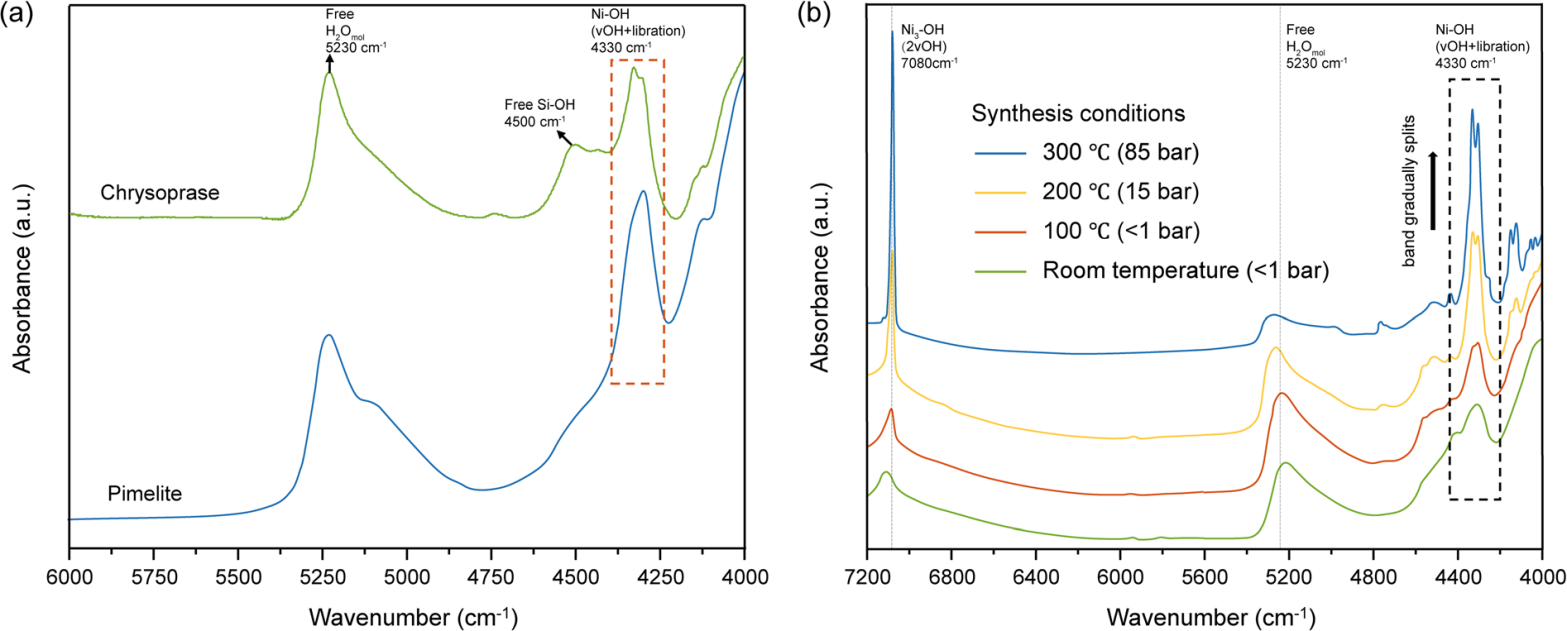
(a) Comparison of NIR spectra between chrysoprase (this study) and pimelite (modified after Reddy *et al.*^52^), highlighting the Ni–OH combination band near 4330 cm^−1^. The chrysoprase spectrum shows band splitting, whereas the pimelite spectrum displays a single, unsplit peak, suggesting differences in local Ni coordination environments. (b) NIR spectra of synthetic Ni-endmember talc (*i.e.*, willemseite, Ni_3_Si_4_O_10_(OH)_2_) prepared under varying temperature and pressure conditions. With increasing temperature and pressure, the crystallinity improves, and the broad 4330 cm^−1^ Ni–OH band gradually narrows and splits, reflecting the emergence of multiple non-equivalent Ni–OH vibrational modes (adapted from Dumas *et al.*^36^).

Two characteristic absorption features in the NIR region are associated with Ni–OH vibrations. The band centered at approximately 7080 cm^−1^ is primarily attributed to the first overtone (2*ν*_OH_) of the Ni_3_–OH stretching vibration, while the sharp absorption feature near 4330 cm^−1^, which exhibits slight splitting, corresponds to a combination vibration involving Ni–OH stretching and librational modes. The intensities of both bands show a very strong positive correlation with Ni content (*ρ* = 0.964, *p* < 0.001), supporting their assignment to Ni-related hydroxyl environments (Fig. 7). Reddy *et al.*^52^ conducted NIR spectroscopy on pimelite and observed a Ni–OH combination band at approximately 4330 cm^−1^. However, unlike chrysoprase, where the 4330 cm^−1^ Ni–OH band exhibits noticeable splitting, the corresponding band in pimelite appears as a single, unsplit feature (Fig. 8a). This difference suggests variations in the local structural environment between the two materials.

**Fig. 9:**
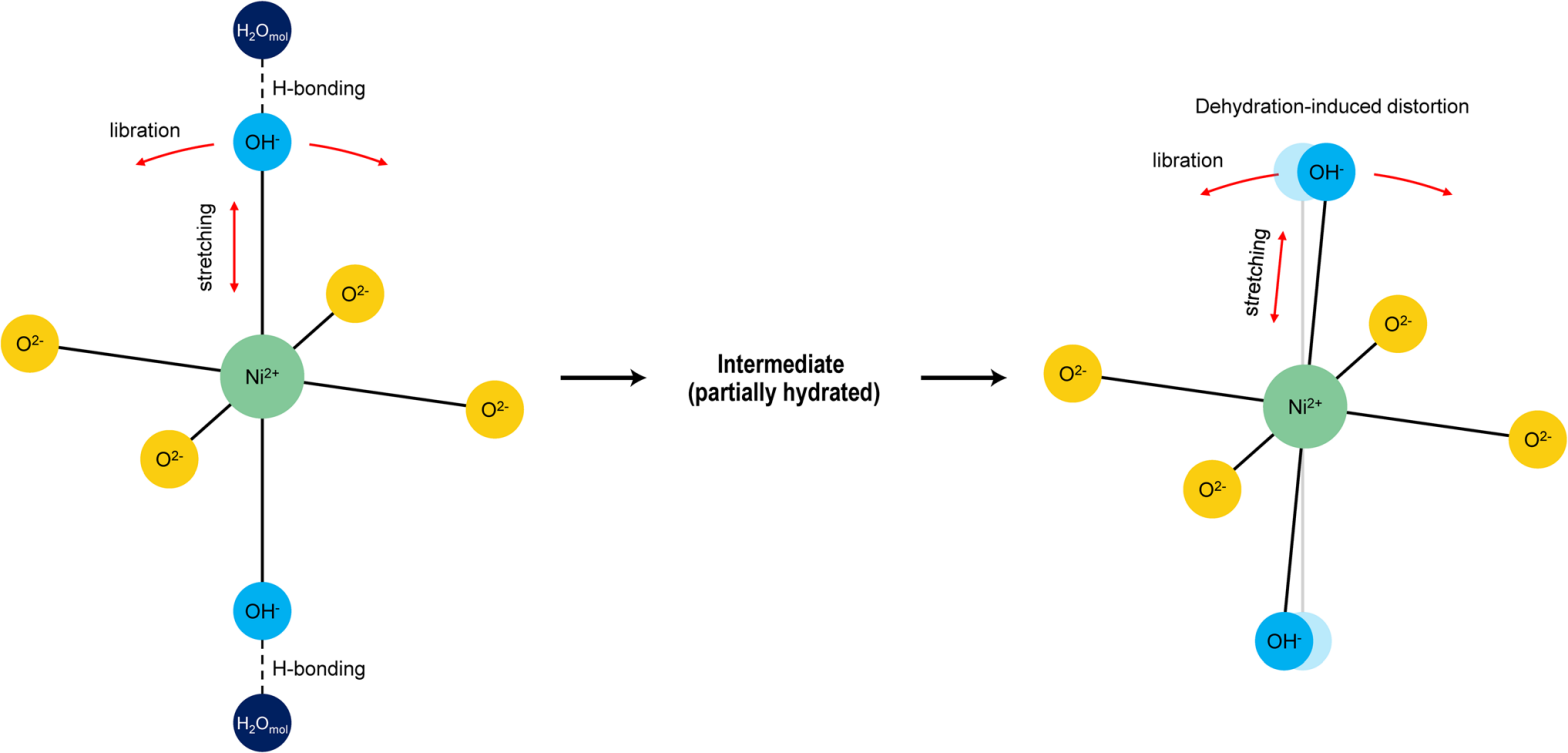
Schematic illustration of the Ni^2+^ coordination environment before (left), during (center), and after (right) dehydration. In the hydrated state (pimelite-like), interlayer water molecules form hydrogen bonds with OH^−^ groups, stabilising the Ni–OH octahedra and maintaining a relatively symmetric crystal field. The intermediate state represents a partially dehydrated condition, in which most Ni–OH groups remain hydrogen-bonded while a fraction lose hydrogen bonding, leading to incipient distortions. In the anhydrous state (willemseite-like), the complete loss of hydrogen bonding induces significant structural distortions, resulting in multiple non-equivalent Ni–OH environments. These variations lead to slightly different vibrational frequencies of the Ni–OH stretching and libration modes, which together contribute to the observed splitting of the ∼4330 cm^−1^ combination band in the near-infrared absorption spectra.

Dumas *et al.*^36^ systematically investigated the structural transformation of synthetic Ni-endmember talc (*i.e.*, willemseite, Ni_3_Si_4_O_10_(OH)_2_) from amorphous precursors to crystalline phases under different temperatures and pressures. During the synthesis and crystallisation process, the near-infrared (NIR) spectral features exhibit systematic changes that reflect variations in the local coordination environment of Ni^2+^ (Fig. 8b). In the early amorphous or poorly crystalline stages, Ni^2+^ ions are typically coordinated by oxygen and hydroxyl groups in a relatively symmetric octahedral geometry. Interlayer water molecules form hydrogen bonds with OH^−^ groups, further stabilising this symmetric environment. Such a balanced local structure results in nearly degenerate stretching and libration vibrational modes, which together give rise to a broad Ni–OH-related absorption band near 4330 cm^−1^. As the synthesis temperature and pressure increase, progressive dehydration removes interlayer water and disrupts the hydrogen bonds, increasing local structural flexibility and reducing the symmetry of the Ni^2+^ coordination environment.^14^ These distortions create multiple, non-equivalent local environments for the Ni–OH bonds, resulting in slightly varied frequencies of the stretching and libration modes (Fig. 9). As a result, the original single combination band at 4330 cm^−1^ splits into two distinct absorption peaks in the near-infrared spectra, and the initially broad band becomes narrower with increasing crystallinity, ultimately exhibiting pronounced splitting. This spectral evolution reflects the structural transition from disordered, hydrous Ni-silicates such as pimelite to well-ordered, anhydrous talc-like phases such as willemseite.

Based on the presence of band splitting at 4330 cm^−1^, which is absent in reported NIR spectra of pimelite but observed in chrysoprase, it can be inferred that the Ni-bearing phase in chrysoprase exhibits a more distorted and less hydrated Ni–OH environment. Additionally, the XRD pattern showing a broad reflection near *d* ≈ 10 Å suggests low crystallinity, making it unlikely to be well-crystallised willemseite. These observations support the interpretation that the colour-causing phase in chrysoprase is a poorly crystalline Ni-bearing phyllosilicate intermediate between disordered, hydrous pimelite and well-crystallised, anhydrous willemseite.

By comparing the 4330 cm^−1^ absorption feature of chrysoprase with the synthetic Ni-endmember talc series reported by Dumas *et al.*,^36^ the degree of peak splitting observed in chrysoprase lies between that of samples synthesised at 100 °C/1 bar and those at 200 °C/15 bar (Fig. 8b). This suggests that the Ni-silicate phase in chrysoprase experienced partial dehydration and structural reorganisation under intermediate conditions. Although not directly measured, these conditions are inferred by analogy with the synthetic reference system^36^ and are plausibly consistent with formation temperatures of approximately 100–200 °C and pressures of about 1–15 bar. Such conditions align with a shallow burial or diagenetic environment, potentially influenced by low-temperature hydrothermal activity, which facilitates the stabilisation of intermediate Ni-phyllosilicate phases such as partially dehydrated pimelite.

## 5 Conclusion

Through a multidisciplinary approach combining colourimetry, XRD, XRF, and NIR spectroscopy, this study reveals that the apple-green colour of chrysoprase is jointly influenced by nickel content and the degree of crystallinity within the silica matrix. In contrast to earlier studies that typically relied on a single line of evidence—such as bulk chemical analyses of Ni content or XRD analyses of structural order—this integrated strategy enables the relationship between composition, structure, and colour to be resolved in a unified framework. The observed correlations among Ni content, crystallinity, and chroma suggest that chroma enhancement in chrysoprase arises from higher abundances of Ni-bearing phyllosilicates, which both increase nickel content and introduce structural disorder within the silica matrix. In this framework, the incorporation of Ni phases disrupts the host lattice order while simultaneously intensifying chroma, thereby linking chemical composition and microstructural disorder in a coupled mechanism. NIR spectral analysis, especially the partially split 4330 cm^−1^ absorption feature, suggests a poorly crystalline Ni-bearing phyllosilicate intermediate between hydrous, disordered pimelite and well-crystallised, anhydrous willemseite.

By linking chemical composition, structural disorder, and colour expression within a single framework, this work not only resolves a long-standing mineralogical debate but also provides broader implications. In gemmology, the clarified mechanism offers objective criteria for distinguishing natural chrysoprase from dyed imitations, since the diagnostic 4330 cm^−1^ absorption feature reflects intrinsic Ni-phyllosilicate environments rather than artificial treatments. In geoscience, the identification of poorly crystalline Ni-phyllosilicates as the colour-causing phase suggests a plausible low-temperature diagenetic formation environment, inferred by analogy with synthetic Ni-silicate reference systems, thereby providing valuable insights into the genesis and provenance of chrysoprase deposits.

## Author contributions

Yuansheng Jiang: conceptualization, methodology, investigation, data curation, formal analysis, visualization, writing – original draft. Qingfeng Guo: supervision, resources, funding acquisition, project administration, writing – review & editing. Yu Wang: software, formal analysis, writing – review & editing, supervision, project administration. Vien Cheung: validation, writing – review & editing. Stephen Westland: methodology, writing – review & editing. Jiayang Han: data curation, formal analysis. Xiang Zong: investigation, resources. Ying Guo: data curation, visualization. Dan Wang: resources, project administration.

## Conflicts of interest

The authors declare that they have no known competing financial interests or personal relationships that could have appeared to influence the work reported in this paper.

## Supplementary Material

RA-015-D5RA05339K-s001

## Data Availability

The data supporting this article have been included as part of the SI, which contains the XRD and NIR spectral data supporting the findings of this study. Supplementary information is available. See DOI: https://doi.org/10.1039/d5ra05339k.
